# Evidence for continuing professional development standards for regulated health practitioners in Australia: a systematic review

**DOI:** 10.1186/s12960-023-00803-x

**Published:** 2023-03-20

**Authors:** Penelope Ann Elizabeth Main, Sarah Anderson

**Affiliations:** 1 Research and Evaluation Team, Australian Health Practitioner Regulation Agency, Melbourne, VIC Australia; 2 School of Allied Health, Human Services and Sport , La Trobe University, Bundoora, VIC Australia

**Keywords:** Continuing professional development, Continuing education, e-learning, Health practitioners, Regulatory standards, Systematic review

## Abstract

**Background:**

Health practitioner regulators throughout the world use continuing professional development (CPD) standards to ensure that registrants maintain, improve and broaden their knowledge, expertise and competence. As the CPD standard for most regulated health professions in Australia are currently under review, it is timely that an appraisal of the evidence be undertaken.

**Methods:**

A systematic review was conducted using major databases (including MEDLINE, EMBASE, PsycInfo, and CINAHL), search engines and grey literature for evidence published between 2015 and April 2022. Publications included in the review were assessed against the relevant CASP checklist for quantitative studies and the McMaster University checklist for qualitative studies.

**Results:**

The search yielded 87 abstracts of which 37 full-text articles met the inclusion criteria. The evidence showed that mandatory CPD requirements are a strong motivational factor for their completion and improves practitioners’ knowledge and behaviour. CPD that is more interactive is most effective and e-learning is as effective as face-to-face CPD. There is no direct evidence to suggest the optimal quantity of CPD, although there was some evidence that complex or infrequently used skills deteriorate between 4 months to a year after training, depending on the task.

**Conclusions:**

CPD is most effective when it is interactive, uses a variety of methods and is delivered in a sequence involving multiple exposures over a period of time that is focused on outcomes considered important by practitioners. Although there is no optimal quantity of CPD, there is evidence that complex skills may require more frequent CPD.

**Supplementary Information:**

The online version contains supplementary material available at 10.1186/s12960-023-00803-x.

## Background

Health practitioner regulators in Australia and other jurisdictions use registration standards to define the requirements that health practitioners need to meet to become, or stay registered. These commonly include standards for primary education in the profession, recency of practice (ROP) and continuing professional development (CPD).

In 2010 Australia introduced the National Registration and Accreditation Scheme (the National Scheme) which regulates 16 health professions under the Health Practitioner Regulation National Law as in force in each state and/territory (the National Law). The National Law requires that National Boards must develop, consult on, and recommend certain registration standards to the Ministerial Council. These core registration standards are generally reviewed by National Boards every 5 years in line with good regulatory practice. The CPD standards for the majority of health professions regulated under the National Law are currently under review. The review of CPD standards requires an understanding of the contemporary evidence base.

CPD has been defined as ‘The systematic maintenance, improvement, continuous acquisition and/or reinforcement of life-long knowledge, skills and competencies of health professionals’[1]. CPD is how health practitioners maintain, improve and broaden their knowledge, expertise and competence, to develop the personal and professional qualities required throughout their professional lives. Health practitioners who are engaged in any form of practice are required to participate regularly in CPD to maintain, update and enhance their knowledge, skills and performance to help them deliver appropriate and safe healthcare.

Research over the past 10 years has demonstrated that while there is good evidence that CPD is effective in increasing practitioner knowledge, there is less evidence that it changes clinical practice, and even less linking it to improved patient safety [2–4]. In 2010, an Institute of Medicine study found major flaws in the way in which CPD was conducted, financed, regulated and evaluated [4] which led to a greater focus on strategies, such as maintenance of certification and other quality assurance activities [5–8]. A CPD mapping exercise conducted in 2015 found considerable variance in CPD standards across European jurisdictions, with a trend toward increased mandatory requirements for CPD and revalidation [1]. As there is no recent multi-profession review of CPD in the literature, it is timely to review the current evidence.

## Aim

The aim of this systematic review is to develop an up-to-date evidence base that will support the development of consistent, evidence-based, effective CPD standards.

## Research questions

1. Is there evidence to support an optimal quantity of CPD to maintain competence? Does the evidence suggest any benefit or disadvantage in requiring CPD to be completed over a particular period, e.g., 1, 2 or 3 years? Is there a case for these to vary between health professions? Or to vary within the same health profession depending on differences in scope of practice/practice division/endorsement?
2. Does the evidence indicate that some types of CPD (including virtual) are more effective at improving practitioner competence and patient safety?
3. Is there evidence to suggest that either self-directed CPD or mandated CPD are more effective at promoting practitioners’ competence and/or patient safety? Should some CPD be mandated? Is a mix of mandated and self-directed CPD more effective? If so, is there an optimal ratio of self-directed to mandated CPD?
4. Is there any evidence that CPD that has been accredited or subject to some quality assurance process is more effective in maintaining clinical competency and/or patient safety outcomes? What factors should be taken into consideration in accrediting CPD?
5. Under what circumstances could an exemption from CPD be justified? Is there evidence to suggest that a short gap in CPD, e.g., 1 or 2 years, has a negative effect on professional competency, including any specific timeframes for this effect to appear?
6. Is there any evidence to suggest a benefit or disadvantage to requiring CPD that is more focused on maintaining a practitioners’ competence in their current scope of practice? What is the evidence about best practice in supporting CPD for practitioners who may wish to change their scope of practice? Is there any evidence to suggest that CPD contributes to other aspects of professional practice?
7. Is CPD more effective when based on a practitioner’s assessment/reflection, peer review or based on curriculum to address their learning needs/skills gap rather than CPD that is based on meeting an externally set requirement, i.e., measured in hours or/points?
8. Should practitioners who hold limited registration (or short-term temporary registration through the pandemic response sub-register) be required to carry out CPD?

## Method

A systematic review was conducted examining the above research questions based on selection criteria, methods and analysis that are summarised below.

The development of the research questions and search terms was informed by two unpublished reviews. These were a commissioned systematic review conducted by the Joanna Briggs Institute in 2012 and an update of that review by the Australian Health Practitioner Regulatory Agency (Ahpra), in 2015.

The full protocol for the systematic review was recently published [9].

### Searching and screening

The search terms and sources of literature selected for the review are based on the experience of the authors in conducting a systematic review of the evidence for CPD standards. The sources included journal articles and grey literature published between 2015 and April 2022, as well as preliminary testing.

#### Search terms

Search terms were selected for the health practitioner group, intervention, and outcome using a combination of the National Library of Medicine Medical Subject Headings (MeSH) and additional relevant search terms. Boolean operators were used to combine terms, and ‘wild cards’ were used to account for plurals and variations in spelling. MeSH is a standardised hierarchically organised vocabulary developed by the National Library of Medicine to index, catalogue and search biomedical and health-related information. The search strategy for this review is presented in Additional file 1: Appendix A and can also be found in the published research protocol [9].

#### Sources of literature

The main sources of literature were:

- Research databases including the Medical Literature Analysis and Retrieval System Online (MEDLINE), Excerpta Medica dataBASE (EMBASE), PsycINFO and Allied and Complementary Medicine Database (AMED) (using the OVID platform) and the Cumulative Index of Nursing and Allied Health Literature (CINAHL)
- Search engines comprising Google Scholar and Google Advanced
- Grey literature produced by other regulatory organisations, government bodies and professional associations
- Reference lists of papers and reports selected for review.

#### Inclusion and exclusion criteria

Articles and reports were included in the systematic review if they met the following criteria:

1. The focus of the report/article is CPD for those health professions regulated under the National Scheme
2. Reviews, original research, reports and theses
3. Published from 1 January 2015 to 30 April 2022
4. Written in the English language
5. For research question 2, must include a comparator group.

Articles and reports were excluded from the systematic review if they met the following criteria:

1. Focussed on health and other professions not regulated under the National Law
2. Focussed on students or interns
3. Focussed on regulatory standards other than CPD
4. Opinion pieces, newsletters, conference presentations
5. Published before 1 January 2015
6. Not written in the English language.

#### Data extraction

Titles identified from the search were checked and the abstract reviewed, where the title appeared to be relevant to the research questions. Where the abstract met the inclusion criteria the full article was downloaded and assessed against the inclusion/exclusion criteria.

A Microsoft Excel spreadsheet was used to record bibliographic information about each article or report (e.g., author, date, title), the study population (e.g., health profession, size, country), intervention (e.g., type of CPD), main findings, study type, the Australian National Health and Medical Research Council level of evidence [10], decisions as to inclusion/exclusion (including any reasons for exclusion) and the quality assessment.

### Quality appraisal

Where the full text of the article was assessed as relevant to the research question(s), a quality appraisal was conducted independently by two people. The published protocol [9] was modified to use the Critical Appraisal Skills Programme (CASP) checklist for systematic reviews [11], which was modified slightly to assess the quality of narrative reviews included in the study. The McMaster University checklists were used for quantitative and qualitative studies [12, 13].

## Findings

### Study selection

Our search strategy identified 20 732 studies through database searching, with an additional 96 records identified through other sources, resulting in 19 517 records after duplicates were removed. Of these, 569 records were screened based on their title and 18 948 records were excluded. Eighty-seven full text articles were assessed for eligibility based on their abstract, of which 49 full-text articles were excluded, because they did not meet the inclusion criteria.

### Description of included studies

Thirty-seven studies were included in the review comprising: 11 systematic reviews (one a meta-analysis), one randomised controlled trial, one quasi-experimental study, four cohort studies, one case–control study, three mixed methods studies, two semi-structured interviews, two correlational studies, four cross-sectional studies, four narrative reviews and four descriptive reviews.

The quality of each study was assessed using either the CASP checklist for systematic reviews [11] or the quantitative [12] or qualitative McMaster checklists [13]. Studies were assessed as of high quality if they addressed all items on the checklist, medium if it lacked one or two components and low if they failed to address more items on the checklist.

The characteristics and quality assessment of the included studies are outlined in Table 1.

**Table 1:** Characteristics and quality assessment of the included studies

Study design	Author	Country	Participants	Study size	Relevant research question(s)	Quality
Systematic review and meta-analysis	Fontaine et al*.* [27]	Canada	Medical practitioners, nurses, health sciences students, mixed health professionals	21 studies *N* = 3543 participants	RQ 2—type of CPD	High
Systematic review	Cant et al. [32]	Australia	Nurses	16 systematic reviews	RQ 2—type of CPD	High
	Cervero and Gains [22]	United States	Medical practitioners	8 systematic reviews	RQ 2—type of CPD	Moderate
	Granchi et al*.* [35]	Australia	Surgeons	19 studies	RQ 2—type of CPD	Medium
	King et al*.* [34]	United Kingdom	Nurses	39 studies	RQ 2—type of CPD	Medium
	Reeves et al*.* [31]	United Kingdom	Podiatrists, complementary therapists, dentists, dieticians, medical practitioners, hygienists, paramedics, psychologists, psychotherapists, midwives, nurses, pharmacists, physiotherapists, occupational therapists, radiographers, speech therapists, social workers, assistant practitioners, care/case co-ordinators and managers	25 studies	RQ 2—type of CPD	High
	Rohwer et al*.* [25]	United Kingdom	Medical practitioners, nurses, physiotherapists, physician assistants, athletic trainers and mixed health professionals	24 studies	RQ 2—type of CPD	High
	Rouleau et al*.* [28]	Canada	Registered nurses	22 systematic reviews	RQ 2—type of CPD	High
	Samuel et al. [23]	United States	Medical practitioners, nurses, dentists, pharmacists, other allied health professionals, mixed professionals	63 syntheses	RQ 2—type of CPD	Medium
	Vaona et al*.* [26]	Canada	Medical practitioners, nurses, childcare health consultants, mixed health professionals	16 RCTs	RQ 2—type of CPD	High
	Vazquez-Calatayud et al*.* [55]	Spain	Nurses	9 studies	RQ 6—scope of practice	Medium
Randomised controlled trial	Mehta et al*. *[58]	United States	Rheumatologists	Intervention arm *N* = 26 Control *N* = 63	RQ 7—self-directed, peer or curriculum	Medium
Quasi-experimental study	Wu et al*.* [30]	Singapore	Nurse preceptors	Intervention arm *N* = 75 Control arm *N* = 75	RQ 2—type of CPD	Medium
Cohort	Kelsch et al*.* [37]	United States	Dental hygienists	Mandated *N* = 764 Not mandated *N* = 998	RQ 3—mandated CPD	Low
	Neimeyer et al*.* [39]	United States	Psychologists	*N* = 790 participants	RQ 3—mandated CPD	Low
	Rothke et al*.* [38]	United States	Psychologists	*N* = 5,215 participants	RQ 3—mandated CPD	Medium
	Vandergrift et al. [14]	United States	Medical practitioners	Policy change *N* = 3,954 No change *N* = 15,609	RQ 1—quantity of CPD	Medium
Case control	Wenghofer et al*. *[40]	Canada	Medical practitioner	Cases *N* = 942 Controls *N* = 1,850	RQ 3—mandated CPD	High
Qualitative–mixed methods	College of Dental Hygienists of British Columbia	Canada	Dental hygienists	Survey *N* = 2,886 Survey of sub-set *N* = 71 Focus groups *N* = 13	RQ 1—quantity of CPD RQ 7—self-directed, peer or curriculum	Medium
	Drumm et al*. *[42]	Republic of Ireland	Pharmacists	*N* = 7 representatives of different accreditation bodies	RQ 4—accreditation	High
	Schindel et al. [51]	Canada	Pharmacists	Focus groups *N* = 42 Survey *N* = 416	RQ 6—scope of practice	Medium
Qualitative–semi-structured interviews	Austin and Gregory [52]	Canada	Pharmacists	*N* = 20 participants	RQ 6—scope of practice	Medium
	Hobbs et al*.* [54]	Australia	Paramedics	*N* = 10 participants	RQ 6—scope of practice	Medium
Correlational	Horn et al. [56]	United States	Paediatric nurses	*N* = 74 participants	RQ 6—scope of practice	Low
	Yardbrough et al*.* [57]	United States	Nurses	*N* = 67 participants	RQ 6—scope of practice	Low
Cross-sectional	Buttars et al*.* [36]	United States	Psychologists	*N* = 294 participants	RQ 2—type of CPD	Low
	Fairs [53]	New Zealand	Osteopaths	*N* = 303 participants	RQ 6—scope of practice	Medium
	Novakovitch [41]	United States	Nurses	*N* = 10 webinars	RQ 4—accreditation	Low
	Salinas et al*.* [29]	United States	Medical practitioners	*N* = 605 CME activities	RQ 2—type of CPD	Low
Narrative review	Atesok et al*.* [18]	United States	Orthopaedic residents	21 studies	RQ 1—skills fade	Medium
	Gawad et al. [19]	Canada	Surgical residents, faculty members	5 cohort studies	RQ 1—quantity of CPD	Medium
	Maddocks et al*.* [17]	New Zealand	Military GPs, ICU/emergency nurses, military and civilian nurses, resident medical officers	10 studies	RQ 1—quantity of CPD	Medium
	Wallace and May [21]	United States	Medical practitioners	62 studies	RQ 2—type of CPD	Medium
Descriptive^a^	Clark [50]	Canada	Occupational therapists	n/a	RQ 6—scope of practice	n/a
	McMahon et al*.* [48]	United States	Medical practitioners	n/a	RQ 4—accreditation	n/a
	Regnier et al. [33]	United States	Medical practitioners	n/a—includes 3 cases	RQ 2—type of CPD	n/a
	Regnier et al*.* [43]	Germany	Medical practitioners	n/a	RQ 4—accreditation	n/a

^a^Critical appraisal of the descriptive studies was not possible; however, these have been included as the content is valuable to the topic area

## Research question 1

### Is there evidence to support an optimal quantity of CPD to maintain competence? 

The systematic review found no direct evidence for an optimal quantity of CPD to maintain the competence of health practitioners. 

Indirect evidence comes from a medium quality cohort study that compares the effect of changes in CPD requirements for physicians in the United States, with their performance in a national maintenance of certification examination [14]. This study found evidence that, those who had been subject to more rigorous CPD requirements performed better in the exam as shown by a change in their results from 50th percentile to 54th percentile. However, the authors were unable to link the findings to improvements in clinical behaviour or patient outcomes.

### Does the evidence suggest any benefit or disadvantage in requiring CPD to be completed over a particular period, e.g., 1, 2 or 3 years?

The review found no evidence to suggest any benefit or disadvantage in requiring CPD to be completed over a particular period. The College of Dental Hygienists of British Columbia extended the duration of their regulatory cycle from 3 to 5 years when they introduced their quality assurance program in 2010 [15]. The length of the registration cycle was increased to balance the additional time and expense that registrants spend doing quality assurance activities. These include completion of an online jurisprudence learning module that includes up-dates of legislation, standards and ethics requirements, self-assessment of learning needs through open book online assessment of knowledge of foundational dental hygiene competencies, development of an online learning plan to address weaker areas, completion of CPD activities and evaluation or reflection on how the CPD activities meet the learning goals and their implementation into practice.

### Is there a case for these to vary between health professions? Or to vary within the same health profession depending on differences in scope of practice/practice division/endorsement? 

There is no evidence for the quantity of CPD to vary between health professions or within the same profession depending on scope of practice/practice division/endorsement. There is also no evidence as to whether CPD is more effective if it is carried out in smaller amounts, more frequently.

There is good evidence, however, that the retention of knowledge and skills varies with the task, such that it would be beneficial for skills to be refreshed more frequently for more complex technical skills, such as surgical or resuscitation procedures, that are practised infrequently [16–19]. For example, emergency airways management and defibrillation skills decrease between 4 and 6 months after training [16, 17], whereas laparoscopic surgical skills decrease 6–8 months after training, and catheter insertion skills for haemodialysis do not decrease until after 1 year [16]. Three studies within a systematic review of CPD for paramedics suggests that there is a ‘dilution’ effect on clinical competence, with long periods of ‘standby’ a major contributor to their de-skilling [20]. For surgical interns, longer breaks between using the skills are associated with greater skill decay [19] and there is a wider range of assessment scores between practitioners who attended the same training sessions [16].

Two qualitative research studies found that medical residents and faculty staff perceive that skills decay is greatest in loss of technical skills, followed by a decrease in knowledge of procedural steps with the extent of perceived skill reduction related to the level of skill difficulty [16, 19].

## Research question 2

### Does the evidence indicate that some types of CPD (including virtual) are more effective at improving practitioner competence and patient safety?

#### Single compared to multi-component approaches to CPD

The systematic review identified three well-designed studies that provide an insight into the efficacy of different types of CPD in improving practitioner competence and patient safety [21–23]. These are two medium quality systematic reviews of syntheses [22, 23] and a medium quality narrative review of systematic reviews [21] that provide consistent evidence that traditional or formal forms of CPD, such as conference presentations, lectures and symposia, taken alone have very little impact on improving clinician performance or patient health outcomes. To illustrate, the table below developed by Wallace and May, 2016, shows the number of systematic reviews reporting high, moderate, low or no effect of common CPD activities on clinical performance and patient outcomes (Table 2) [21].

**Table 2:** Effect of traditional CPD activities on clinical performance and patient outcomes (Wallace and May 2016, adapted from Bloom 2005)

CPD activity	Effect on clinician performance				Effect on patient outcomes			
	High	Moderate	Low	None	High	Moderate	Low	None
Didactic programs	0	3	7	10	0	0	0	14
Interactive	5	6	2	0	0	3	1	3
Audit/feedback	6	11	4	2	0	5	3	2
Academic outreach	6	8	1	0	1	4	1	0
Opinion leaders	0	3	4	2	1	0	0	0
Reminders	9	9	5	0	2	4	2	2
Clinical practice guidelines	0	3	2	0	0	1	0	0
Information only	0	2	3	8	0	0	1	2

It can be seen from the table that didactic programs alone have very little impact on clinician performance or patient outcomes [21], although they have been shown to improve practitioner knowledge [22]. All three reviews showed that CPD leads to greater improvement in health practitioner performance and patient outcomes if it is more interactive, uses a variety of methods (such as academic detailing, case-based learning, demonstrations, feedback, lectures, problem-based learning, point-of-care techniques, role play and patient simulations) and is delivered in a sequence to the learner involving multiple exposures over a longer period of time and is focused on outcomes that are considered important by practitioners [21–23]. These findings are in line with those of two previous systematic reviews conducted before the study period [2, 24].

Recent research looking at the impact of CPD on health practitioners’ (medical practitioners, nurses, dentists, pharmacists and allied health professionals) clinical performance and/or patient outcomes found that of 63 syntheses included in the review, 38 (60%) included multi-component approaches, and 29 (46%) incorporated e-learning approaches, either singly or in combination with other interventions [23]. While 42 (67%) syntheses reported outcomes affecting healthcare practitioners’ behaviour change and/or patient outcomes, most of the findings reported for patient outcomes were not statistically significant. 

#### Electronic learning

The systematic review identified a low quality meta-analysis of outcomes assessments of CPD, as well as four high quality systematic reviews, one a meta-analysis, and a medium quality quasi experimental study that compare an electronic learning (e-learning) approach with traditional learning for health professionals [25–30]. The findings vary widely; however, they consistently show that e-learning is as effective as face-to-face CPD approaches.

The meta-analysis of outcomes assessments of around 600 CPD programs for medical practitioners in the United States found that, in general, online activities (such as interactive text, case-based and multimedia activities) were more effective than live activities (such as dinner meetings, symposia or workshops) and both were more effective than ‘enduring’ activities (such as provision of printed materials, mobile text or audio) [29]. Mobile content was less effective than other formats. The outcomes assessments were collected by an independent educational research company over 9 years. There were insufficient details about the methods to assess the risk of bias.

Fontaine et al.’s (2019) systematic review and meta-analysis of 21 studies (representing 3 684 participants) found that personalised e-learning significantly improved clinical skills compared to traditional forms of training [27]. However, the authors note that caution should be exercised in interpreting the findings because of the high degree of heterogeneity between studies.

A Campbell Collaboration systematic review of 24 trials (representing 3 825 participants) found good evidence that a blended e-learning approach to training for evidence-based healthcare leads to a greater improvement in attitudes and behaviours than either face-to-face training or pure e-learning [25]. The study found no difference in effectiveness between e-learning or face-to-face learning compared to no training. The authors note that caution should be exercised in interpreting the findings as many of the studies included were small and subject to bias.

A quasi-experimental study that examined the effectiveness of a web-based pedagogy program for nurse preceptors compared to face-to-face teaching found that, although participants of the face-to-face teaching program had significantly higher scores for clinical teaching and assessment behaviour in clinical settings immediately after the program, there was no difference in outcome between the groups 6 months later [30].

The findings of the other three systematic reviews that compare e-learning to traditional learning were inconclusive [26–28]. The first is a Cochrane Collaboration review that includes 16 randomised controlled trials of e-learning (representing 5 679 participants) compared to traditional learning approaches (defined as any mode of learning other than e-learning) for screening and treatment of high cholesterol [26]. Compared to traditional learning at 12-month follow-up, e-learning made little or no difference to patient outcomes as measured through blood tests. At 3–12-month follow-up, there was little or no difference in clinical behaviour regarding screening or treatment of high cholesterol, and it was uncertain whether e-learning made any difference to skills or knowledge in the first 3 months after training.

Finally, Rouleau et al*.* 2019 considered 22 systematic reviews that examined the efficacy of e-learning CPD activities for nurses [28]. The systematic reviews included in the study were very heterogeneous. Types of e-learning interventions included online and interactive CD-ROM programs, computer-based simulations, and video-conferencing and comparators varied from face-to-face presentations to blended learning to no intervention. Nurses reported satisfaction with e-learning compared to traditional methods in eight of the 22 systematic reviews (36%) and dissatisfaction in three systematic reviews (14%) mostly because of technical difficulties or lack of familiarity with computers. Increased knowledge was reported in 13 of the 22 systematic reviews (59%) and seven systematic reviews (32%) reported no change in knowledge. None of the systematic reviews reported changes in clinical behaviour, and only one systematic review reported a perceived positive change in patient outcomes. The authors concluded that it remains unknown how effective e-learning is in improving clinical behaviours and patient outcomes.

#### Interprofessional continuing education

The systematic review identified two systematic reviews and a case series that examined the effectiveness of interprofessional CPD [31–33]. A high quality systematic review found there was a significant increase in new studies focusing on inter-professional CPD rather than inter-professional education for undergraduates, up from 29% (six of 21 studies) in 2007 to 48% (12 of 25 additional studies) in 2016 [31]. The authors identified inter-professional coaching, mentoring, and the use of reflection and other informal learning processes as important factors in improving clinician behaviour, practice organisation and patient outcomes. More positive outcomes were reported than mixed, neutral or negative outcomes, particularly where the outcome was the learners’ reaction to the training (25 studies) or changes in knowledge and skills (19 studies). Only 13 studies reported on outcomes related to changes in organisational practice, and 10 studies on patient outcomes. None of the studies reported a negative outcome. Unfortunately, the analysis did not separate the studies related to inter-professional CPD from those that focussed on inter-professional education of undergraduates. The authors concluded that learners respond well to inter-professional coaching, their attitudes and perceptions of one another improve, and they report increases in collaborative knowledge and skills. Evidence related to changes in behaviour, organisational practice and benefits to patients/clients was more limited but growing.

The other high quality systematic review reported that simulation-based methods were widely used for inter-professional team-based CPD that included nurses, and were often combined with other learning modalities, such as web-based information or workshops [32]. It found that there was good evidence that simulation for multi-disciplinary teams has a positive impact on nurses learning; however, only a small proportion of studies included in the review included objective measures of the impact of inter-professional team-based CPD using simulation on clinical practice and/or patient outcomes. These studies reported improved emergency team response, better service delivery (e.g., higher adult and paediatric cardiopulmonary resuscitation survival rates, development of a safety policy, changing equipment), and, better on-site team performance (e.g., faster times to commence patient investigations, reduced mobidity and mortality in perinatal emergencies), 

The case series published by a joint accreditation board in the United States lends further support to the above findings [33]. For example, interprofessional team-based CPD using simulation to teach health-care teams how to care for children with heart disease presenting to the emergency ward of a children’s hospital was shown to lead to changes in protocols, processes and procedures, that resulted in increased use of the hospital emergency care system and the inclusion of electrocardiogram technicians in the care team.

#### Other evidence

The systematic review also identified two medium quality systematic reviews and a low quality cross-sectional study that provide further insights into the efficacy of different types of CPD [34–36]. The first systematic review found that nurses have a strong preference for CPD in the workplace [34]. Factors that optimised the impact were: self-motivation, relevance to practice, strong enabling leadership and a positive workplace culture. Whereas the second found that studies examining the efficacy of group coaching of surgeon’s non-technical skills (i.e., cognitive and interpersonal skills) were more likely to report improvements in performance and patient outcomes than those examining the efficacy of individual coaching [35]. It concluded that there is strong evidence that non-technical skills improve when surgeons are coached in a group setting; however, evidence for individual coaching of non-technical skills for surgeons was limited.

Finally, a low quality cross-sectional study of psychologists 2 months after attending the American Psychology Association’s 2019 continuing education sessions and workshops found that longer CPD sessions (half to full day) and experiential approaches to instruction led to improved knowledge and translation of learning into practice compared to short CPD sessions (1–2 h) [36].

## Research question 3

### Is there evidence to suggest that either self-directed CPD or mandated CPD are more effective at promoting practitioners’ competence and/or patient safety? 

The systematic review identified three cohort studies [37–39] (one medium and two low quality), and a high quality case–control study [40] that reached slightly different conclusions as to whether self-directed CPD or mandated CPD is more effective in promoting practitioners’ competence and/or patient safety. A national survey of dental hygienists in the United States found that the proportion of respondents who complied with infection control guidelines was significantly higher in states that had a mandatory CPD requirement related to infection control, compared to those from states without a mandatory requirement [37].

These findings are supported by a case control study that compares CPD data from medical practitioners (n = 2 792) who received a complaint with the Ontario regulatory body between 2002 and 2003, with those who had not [40]. Using multivariate logistic regression, the authors found that medical practitioners who participated in CPD activities were significantly less likely to have been the subject of a quality of care-related complaint than those who did not.

A longitudinal study showed that the rate of complaints to the regulator for psychologists in Illinois (total number of complaints divided by the number of psychologists during each licensure cycle) remained constant before and after the introduction of mandatory CPD [38]. The average complaint rate during the three licensing cycles before the complete implementation of mandatory CPD (2009–2014) was almost identical to the average rate of complaints for the two licensing cycles following the mandate (2015–2018). Mandatory CPD was introduced over three consecutive registration years (2012 and 2015) which makes the findings harder to interpret. Following the implementation of mandatory CPD, psychologists in Illinois reported a significant increase in perceived learning and clinical effectiveness [39]; however, these changes were not measured objectively.

### Should some CPD be mandated? Is a mix of mandated and self-directed CPD more effective? If so, is there an optimal ratio of self-directed to mandated CPD?

As identified above, a single study above showed that those who did mandated CPD training were more compliant with guidelines than those who did not [37].

## Research question 4

### Is there any evidence that CPD that has been accredited or subject to some quality assurance process is more effective in maintaining clinical competency and/or patient safety outcomes?

The systematic review identified a low quality pilot study that used a matrix based on the American Nurses Credentialing Center Primary Accreditation Criteria to determine whether learners observed differences in the quality of educational activities developed by organisations using accreditation criteria, compared with those who did not [41]. The study found a measurable learner perceived difference that the quality of CPD was higher for activities developed by organisations using accreditation criteria, compared with those that did not. They concluded that accreditation criteria are an effective way of identifying high quality educational activities that are designed to positively influence practice and patient outcomes, although the study did not link accreditation to patient outcomes per se. Caution needs to be exercised when making conclusions based on this study due to the lack of independence of the assessment and lack of rigour in the study design.

### What factors should be taken into consideration in accrediting CPD?

An evidence-based accreditation framework was developed by the Global Forum on Quality Assurance of Continuing Education and Continuing Professional Development^1^ for pharmacy CPD across international jurisdictions [42]. The process was based on the findings of a literature review, a survey of accreditation processes in the countries represented by member of the forum. This was followed by four rounds of a Delphi consensus process resulting in agreement of 15 items across four stages (Input, Process, Output, and Quality Improvement) of CPD accreditation:

- Accreditation inputs (context for activity, accreditation standards/processes, quality processes, educational content, method of delivery, assessment approach, evaluation of the activity, impact of the activity, reflective practice)
- Accreditation process (application process and application review process)
- Accreditation output (decision and appeals process)
- Quality improvement (review of activity and evaluation by participants).

**Fig. 1:**
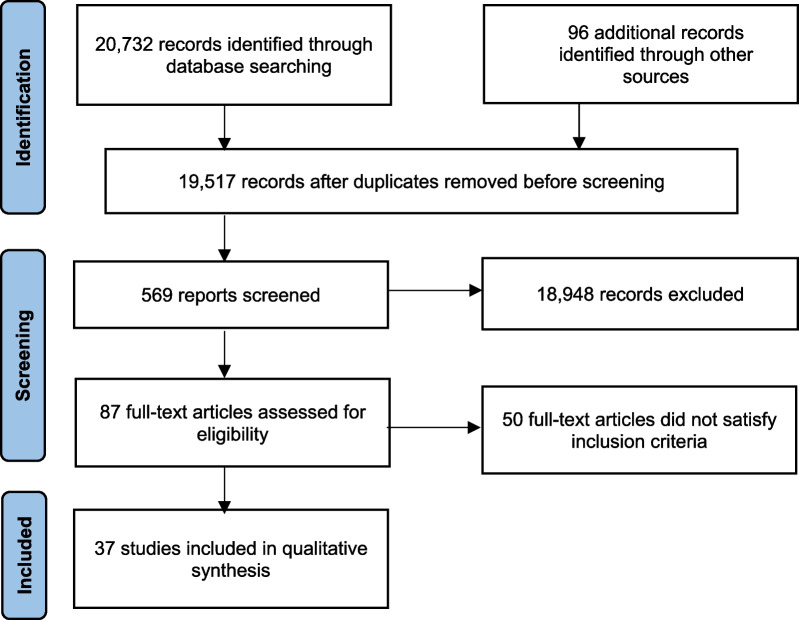
PRISMA Flowchart of studies included in the systematic review

A similar process was used to develop a shared set of international standards for the accreditation of CPD for medical practitioners and health-care teams [43]. The standards fall into six domains: eligibility and responsibility of the accrediting body, independence and transparency in accredited education, needs assessment used for planning, content validity, quality of educational design, assessment of the impact on the learning/competence of medical practitioners and/or the health status of their patients (see Fig. 1).

Several authors have recommended that the provision of CPD should be independent of any potential conflicts of interest between pharmaceutical companies or commercial suppliers and health professionals [44–47]. There has been some debate as to whether accreditation protects against sponsored CPD [48, 49].

## Research question 5

### Under what circumstances could an exemption from CPD be justified?

The systematic review did not find any evidence to support any circumstances under which an exemption from CPD could be justified. 

### Is there evidence to suggest that a short gap in CPD, e.g., 1 or 2 years, has a negative effect on professional competency, including any specific timeframes for this effect to appear?

There is no direct evidence to suggest that a short gap in CPD has an effect on professional competency. The limited evidence that we have on skills fade suggests that we cannot make an evidence-based recommendation for exemptions from CPD.

## Research question 6

### Is there any evidence to suggest a benefit or disadvantage to requiring CPD that is more focused on maintaining a practitioners’ competence in their current scope of practice?

There was no evidence to suggest a benefit or disadvantage to requiring CPD that is more focused on maintaining a practitioners’ competence in their current scope of practice.

### What is the evidence about best practice in supporting CPD for practitioners who may wish to change their scope of practice? 

The systematic review was unable to identify any evidence about the best way to support CPD for practitioners wishing to change their scope of practice. The review identified a paper outlining CPD support provided by a Canadian college for occupational therapists wishing to change their scope of practice [50], and two medium quality qualitative research studies regarding CPD to support the broadening of scope of pharmacy practice [51, 52]. The qualitative research studies were complementary, with one identifying the topics pharmacists felt they needed additional training on and both studies showing a preference for more interactive forms of learning.

The College of Occupational Therapists of British Columbia has implemented support for registrants who are changing scope of practice through their two-part annual continuing competency review [50]. The first part of the annual review focusses on current practice and the identification of any transitions that may affect their competence using a standardised list of transitions and supports to help in this process. The second part of the review is a practice quiz designed to make registrants aware of potential changes in scope resulting from newly released practice standards or changes in legislation. Registrants can then select CPD activities to support adaptation to the new requirements.

Qualitative research into CPD support for the expanding scope of practice for pharmacists comprised a mixed methods study of pharmacists in Alberta, Canada and semi-structured interviews in Ontario, Canada [51, 52]. In Alberta, where expanded roles for pharmacists include authorisation to prescribe and/or inject drugs, medication review and physical assessment, a web-based survey of 416 pharmacists found that a high proportion wanted training in physical assessment (86%), interpreting laboratory tests (73%) and making decisions about complex drug therapy (72%). Pharmacists included in the survey preferred to learn with peers (78%) and/or within teams in the workplace (71%) [51]. Focus groups conducted with a sub-group of pharmacists (n = 42) revealed that they want to select activities relevant to their desired focus of practice with a greater emphasis on skills development than theoretical knowledge with a preference for face-to-face methods including meetings, mentoring and skills workshops.

The second was a well-designed qualitiative study that comprised semi-structured interviews of 20 community pharmacists in Ontario, Canada to determine their CPD needs in respect of the expanding scope of practice for pharmacists [52]. Seven educational techniques were identified as being most helpful to promote practice change: (i) a coaching/mentoring approach instead of traditional lectures/didactic presentations, (ii) practice-based experiential learning rather than a classroom or web-based delivery, (iii) a longitudinal, incremental approach to instructional design rather than a one-off delivery, (iv) active demonstration of how to implement practice change, (v) increased focus on soft-skills development, such as conflict management, motivational interviewing, and interprofessional collaboration, (vi) opportunities for practice/rehearsal of new skills, and (vii) use of a 360-degree feedback model.

### Is there any evidence to suggest that CPD contributes to other aspects of professional practice?

Evidence to suggest that CPD contributes to other aspects of professional practice is limited. Almost two-thirds of respondents to a national survey of osteopaths registered in New Zealand in 2016 reported that their communication skills improved as a result of CPD (62%), and over half indicated that CPD helped them develop their business (56%) [53]. Semi-structured interviews of Australian paramedics revealed that they perceive CPD as contributing to opportunities for career progression [54]. Similar findings were obtained from a systematic review of CPD and nursing [55] and a correlational study of paediatric nurses in the United States [56]. 

Nurses and paramedics also identified personal fulfilment, improved self-esteem and self-confidence during practice as being another important outcome of CPD [54–56]. A study of nurses working in a medium-sized hospital in the southwestern United States found a weak correlation between job satisfaction and career development with staff retention [57].

There was no evidence for the contribution of CPD to other aspects of professional practice, such as maintaining links with the wider professional community, ethical practice or cultural competency.

## Research question 7

### Is CPD more effective when based on a practitioner’s assessment/reflection, peer review or based on curriculum to address their learning needs/skills gap rather than CPD that is based on meeting an externally set requirement, i.e., measured in hours or/points?

There is limited evidence to suggest that CPD is more effective when based on a practitioner’s assessment/reflection, peer review or based on curriculum to address their learning needs/skills gap rather than CPD that is based on meeting an externally set requirement.

A well-designed medium quality study comparing the effectiveness of CPD that was personalised following standardised assessment to self-directed CPD showed there is greater knowledge translation using a personalised CPD learning path [58]. Rheumatologists were randomly allocated into one of two arms of the study. The first comprised an online assessment to objectively identify specific needs that mapped incorrect responses to one of three activities (n = 26), whereas the second allowed participants to select any combination of the same three activities (n = 63). Rheumatologists who participated in the directed learning path were 36% more likely to make evidence-based choices based on the content of the CPD after participating in the program.

An evaluation of an assessment tool implemented by the College of Dental Hygienists of British Columbia designed to guide registrants in their selection of CPD showed that although there was an increase of 9% in the number of registrants since its introduction in 2012 until the evaluation in 2017, there was no change in the number of complaints in the same period [15]. The tool is a 75 question, open book assessment taken online that provides feedback to registrants on their knowledge of foundational dental hygiene competencies. The tool was introduced to counter poor self-assessment of learning needs and other factors, such as selecting CPD based on convenience. 

## Research question 8

### Should practitioners who hold limited registration (or short-term temporary registration, through the pandemic response sub-register) be required to carry out CPD?

The systematic review was unable to find any evidence as to whether practitioners who hold limited or short term temporary registration should be required to carry out CPD.

## Conclusions and discussion

Australian and international health practitioner regulators have specific CPD requirements to ensure that registrants who are actively engaged in practice regularly participate in CPD that is relevant to their scope of practice. Practitioners carry out CPD to maintain, update and enhance their knowledge, clinical skills and performance to help them deliver appropriate and safe care. In Australia, National Boards set mandatory CPD requirements, although the specifics vary between health professions.

### Mandatory CPD increases participation and has many benefits

Mandatory CPD requirements are a strong motivational factor for the completion of CPD activities [53, 54, 59, 60]. Comparisons of CPD participation in mandated and non-mandated jurisdictions show that psychologists complete one-third fewer CPD credits than their mandated counterparts [61] and physiotherapists complete 16% less [62]. This finding is supported by longitudinal studies that show increased participation in CPD following the introduction of mandatory CPD requirements for psychologists [39] and dental practitioners [63].

The benefits of CPD are that it improves practitioners’ knowledge and behaviour, and evidence for improvement in clinical skills and patient outcomes is growing [2, 22, 24]. Other benefits include improved communication and business skills [53], improved self-confidence and self-esteem, career progression [54–56], and better workforce retention [57]. Therefore, making CPD mandatory has many benefits.

### Ensuring CPD is effective

The systematic review found good evidence that CPD is most effective when it is more interactive and uses a variety of methods, delivered in a sequence involving multiple exposures over a period of time that is focused on outcomes considered important by practitioners [2, 22–24]. The review was unable to find any evidence for an optimal amount of CPD to be completed, although there is good evidence that the retention of knowledge and skills varies with the task. Skills fade is greater for more complex technical skills, such as surgical or resuscitation procedures, if practised infrequently [16–19].

In Australia, CPD registration standards typically require health practitioners to complete a number of CPD hours over a defined period of time. Registrants are often encouraged to reflect on their practice to identify their own developmental needs, carry out the appropriate CPD activities to meet these identified needs, and reflect on how the learnings can be applied in practice, while identifying any further developmental needs. 

This is in line with international best practice; however, many jurisdictions embed their CPD requirements in broader quality assurance initiatives. For example, the College of Dental Hygienists of British Columbia help their registrant's selection of CPD activities with a self-assessment tool designed to identify areas of practice that may benefit from additional training as well as requiring annual completion of a jurisprudence module that provides information on legislation, standards and guidelines relevant to practice [15].

As noted above, the primary goal of CPD requirements is to ensure health practitioner competence and public safety which has led to a call to use competence as an outcome measure [21, 64, 65]. To this end, medical practitioners in the United States and Canada are required to supplement their CPD with 4-yearly maintenance of certification examinations, and in the United Kingdom they are required to undergo a revalidation process through which they must periodically demonstrate their continued fitness to practise [8]. A comprehensive literature review of the best practice in the assessment of competence was published by Newcastle University, United Kingdom in 2018 [66].

### CPD needs to be protected from commercial interests

Researchers have highlighted the need to ensure that the CPD is provided independently of conflicts of interest between pharmaceutical companies or commercial suppliers and health professionals [44–47]. There has been some debate as to whether accreditation protects against sponsored CPD [48, 49]. In Australia, optometry is the only health profession that sets a limit on the quantity of sponsored CPD activities, including CPD activities related to optical goods and equipment, that can be claimed toward the annual CPD requirement. 

### Interprofessional education

A series of Institute of Medicine reports demonstrating the relationship between poor team performance and negative patient outcomes called on accreditors and regulating bodies to introduce policies designed to bring about change [4, 67, 68]. This systematic review found good evidence that inter-professional CPD improves health professionals’ knowledge, behaviour and clinical skills [31, 69], as well as patient and system outcomes [31]. European research suggests that enablers of inter-professional CPD include involving patients in their design and delivery, providing a holistic focus, using multi-modal learning formats, including multiple professions, evaluating formative and summative aspects, and encouraging team-based learning [70]. In the United States, a joint accreditation for inter-professional continuing education body has been established to provide an opportunity for simultaneous accreditation across health practitioner groups [71].

### E-learning and the globalisation of CPD

Apart from one study of uncertain quality that reported better outcomes for e-learning compared to face-to-face learning or use of text-based learning [29], this systematic review found good quality evidence that there is little to no difference between e-learning and other CPD approaches [25–28]. CPD using an e-learning approach has increased during the pandemic [72–76] and is also an important strategy for rural practitioners [77, 78].

The globalisation of access and provision of CPD which increases the availability of e-learning [79–81] has led to initiatives aimed at harmonisation and mutual recognition of CPD standards, particularly in medicine and pharmacy [43, 82, 83]. The Global Forum on Quality Assurance of Continuing Education and CPD, comprising representatives of pharmacy accreditation organisations from Australia, Canada, Ireland, New Zealand, South Africa, the United Kingdom and the United States, developed an accreditation framework for pharmacists [42] and is currently developing strategies to implement it as a means of recognising CPD across boundaries [82]. A similar collaborative group, the International Academy for Continuing Professional Development Accreditation, has developed a shared set of international standards to guide the accreditation of CPD and determine substantive equivalency between accreditation bodies [43]. Currently the standards are focussed on medical practitioners and/or healthcare teams that include medical practitioners. European accreditation bodies have been slower to initiate a similar process; however, an association of accreditors has been formed with the aim of reaching consensus on the principles, rules and practice of CPD as well as its accreditation [83].

### Limitations

The main limitation for the systematic review was the lack of well-designed studies that address most of the research questions. Although there is a very large literature on the efficacy of different types of CPD, most focused on profession-specific issues that could not easily be generalised and most did not include a comparator group. There was a high degree of heterogeneity among these studies which varied widely in the characteristics of the study population and measured outcomes. Older studies relied on self-reported participant reactions or learning to gauge the effectiveness of CPD, whereas newer studies have started to use objective changes in behaviour and/or patient outcomes in their place. The lack of studies using newer objective outcomes is a further limitation of the review. The sample size for the majority of the studies were appropriate for the type of study.

Non-English language articles were excluded, so there is a risk that relevant articles written in another language were not included in this analysis. Another limitation is that the systematic review only included articles published between 2015 to April 2022, which may have excluded important earlier work relevant to the research questions.

### Conclusions

This systematic review found CPD is most effective when it is interactive, uses a variety of methods and is delivered in a sequence involving multiple exposures over a period of time that is focused on outcomes considered important by practitioners. No direct evidence was found for an optimal quantity of CPD required to maintain competence, although there is some evidence that complex or infrequently used skills deteriorate between 4 months to a year after training, depending on the task.

In comparable jurisdictions, there is a move toward output focussed CPD requirements that are embedded in a broader strategy that is designed to ensure competence, as well as increasing recognition of the need for inter-professional education targetting health-care teams and increasing acceptance of e-learning. These issues will benefit from consideration in the current review of National Board CPD registration standards.

## Supplementary Information

**Additional file 1.** Appendix A: CPD search strategy.

## Data Availability

The data and materials are listed in the reference list.

## Footnotes

1 Australia, Canada, New Zealand, Republic of Ireland, South Africa, United Kingdom and United States.
