# Supplementary material for: Evidence for continuing professional development standards for regulated health practitioners in Australia: a systematic review
Source: Hum Resour Health. 2023 Mar 20;21:23. doi: 10.1186/s12960-023-00803-x (PMC10026429; doi:10.1186/s12960-023-00803-x)
Supplement: Supplementary file 1 — Additional file 1. Appendix A: CPD search strategy. [file 12960_2023_803_MOESM1_ESM.docx]

**Appendix A**

| **MEDLINE** |  |
| --- | --- |
|  | Exp Health personnel/ OR health care profession*.ti,ab OR Chinese adj2 herbal*,ti,ab OR ((Chinese med*)adj2 prac*) OR chiropract*.ti,ab OR dent*.ti,ab OR doctor*.ti,ab OR emergency adj2 medical technician OR general practitioner*.ti,ab OR (Exp health workforce/ AND Indigenous AND Australia) OR medica*.ti,ab OR midwif*.ti,ab OR nurs*.ti,ab OR occupational therap*.ti,ab OR optom*.ti,ab OR osteopath*.ti,ab OR paramedic*.ti,ab OR pharm*.ti,ab OR physician*.ti,ab OR physiotherap*.ti,ab OR physical therapist*.ti,ab OR podiatr*.ti,ab OR psycholo*.ti,ab OR radiograph*.ti,ab |
| AND | Exp Competency based education/ OR Exp Education, continuing/ OR Exp education, distance/ OR Exp education, medical, continuing/ OR Exp education, nursing, continuing/ OR Exp education, pharmacy, continuing/ OR Exp educational measurement/ OR Exp learning/ OR Exp peer review, health care/ OR Exp self-assessment/ OR Exp staff development/ OR accredit*.ti.ab OR competency adj2 framework.ti.ab OR competency adj2 standards.ti.ab OR mentoring.ti.ab OR practice adj2 portfolio.ti.ab OR reflective adj2 practi*.ti.ab OR revalidation.ti.ab |
| AND | Exp competence, clinical/ OR Exp competence, professional/ OR Exp cultural competency/ OR Exp inappropriate prescribing/ OR Exp licensure/ OR Exp malpractice/ OR Exp mandatory reporting/ OR Exp patient safety/ OR problem behavior/ OR Exp professional practice/ OR Exp professionalism/ OR Exp quality of health care/ OR Exp scope of practice/ OR advanced ad2j practice.ti.ab OR authentic adj2 learning.ti.ab OR endorsement.ti.ab OR extended adj2 practice.ti.ab OR fitness adj3 practice.ti.ab OR knowledge adj2 transfer.ti.ab OR impaired adj2 practice.ti.ab OR non-medical adj2 prescribing.ti.ab OR registration adj2 standards.ti.ab |
| AND | Limit to yr=2015– April 2022 |
| AND | Limit to English language |
| **Embase** |  |
|  | Exp Health personnel/ OR health care profession*.tw OR Chinese med* adj2 prac* OR chiropract*.tw OR dent*.tw OR doctor*.tw OR general practitioner*.tw OR (Exp health workforce/ AND Indigenous.mp AND Australia.mp) OR medica*.tw OR midwif*.tw OR nurs*.tw OR occupational therap*.tw OR optom*.tw OR osteopath*.tw OR paramedic*.tw OR pharm*.tw OR physician*.tw OR physiotherap*.tw OR physical therapist*.tw OR podiatr*.tw OR psycholo*.tw OR radiograph*.tw |
| AND | Exp Accreditation/ OR Exp Certification/ OR Exp Competency based education/ OR Exp Education, continuing/ OR Exp education, distance/ OR Exp education, medical, continuing/ OR education, nursing, continuing/ OR Exp education, pharmacy, continuing/ OR Exp educational measurement/ OR Exp learning/ OR Exp peer review, health care/ OR Exp self-assessment/ OR Exp staff development/ OR competency adj2 framework.mp OR competency adj2 standards.mp OR mentoring.mp OR practice adj2 portfolio.mp OR reflective adj2 practi*.mp OR revalidation.mp |
| AND | Exp competence/ OR Exp inappropriate prescribing/ OR Exp licensure/ OR Exp malpractice/ OR Exp mandatory reporting/ OR Exp patient safety/ OR Exp problem behavior/ OR Exp professional practice/ OR Exp professionalism/ OR Exp health care quality/ OR scope of practice.mp OR advanced adj practice.mp OR authentic adj2 learning.mp OR endorsement.mp OR extended adj2 practice.mp OR fitness adj3 practice.mp OR knowledge adj2 transfer.mp OR impaired adj2 practice.mp OR non-medical adj2 prescribing.mp OR registration adj2 standards.mp |
| AND | Limit to yr=2015– April 2022 |
| AND | Limit to English language |
| **PsycInfo** |  |
|  | Exp Allied health personnel/ OR Exp Health care profession/ OR Exp Health personnel/ OR Exp Acupuncturist/ OR Exp Dentists/ OR (Exp Health care services AND Exp Indigenous populations/ AND Australia) OR Exp Medical personnel/ OR Exp Midwifery/ OR Exp Military medical personnel/ OR Exp Nurses/ OR Exp Occupational Therapists/ OR Exp Optometrists/ OR Exp Osteopathic Medicine/ OR Exp Paramedical personnel/ OR Exp Pharmacists/ OR Exp Physical therapists/ OR Exp Physicians/ OR Exp Podiatrist OR Exp Psychiatric hospital staff/ OR Exp Psychologists/ OR Exp Radiographer/ OR Chiropractor.mp OR Chinese adj medicine practitioner.mp |
| AND | Exp Competency based education/ OR Exp Education, continuing/ OR Exp education, distance/ OR Exp education, medical, continuing/ OR education, nursing, continuing/ OR Exp education, pharmacy, continuing/ OR Exp educational measurement/ OR Exp learning/ OR Exp peer review, health care/ OR Exp self-assessment/ OR Exp staff development/ OR accredit*.ti.ab OR competency adj2 framework.ti.ab OR competency adj2 standards.ti.ab OR mentoring.ti.ab OR practice adj2 portfolio.ti.ab OR reflective adj2 practi*.ti.ab OR revalidation.ti.ab |
| AND | Exp competence/ OR Exp cultural competency/ OR inappropriate prescribing.mp OR Exp malpractice/ OR mandatory reporting.mp OR Exp medical malpractice/ OR Exp patient safety/ OR Exp prescribing (drugs) OR problem behavior.mp OR Exp professional competence/ OR professional practice.mp OR Exp professionalism/ OR quality of health care.mp OR scope of practice.mp OR advanced adj practice.mp OR authentic adj2 learning.mp OR endorsement.mp OR extended adj2 practice.mp OR fitness adj3 practice.mp OR knowledge adj2 transfer.mp OR impaired adj2 practice.mp OR non-medical adj2 prescribing.mp OR registration adj2 standards.mp |
| AND | Limit to yr=2015– April 2022 |
| AND | Limit to English language |
| **CinAHL** |  |
|  | MH Health personnel OR MH Allied Health Personnel OR MH Physicians OR MH Nurses OR MH Registered nurses OR MH Midwifery OR MH Chiropractic OR MH Dentists OR MH Emergency Medical Technicians OR First National of Australia OR MH Occupational Therapists OR MH Osteopaths OR MH Optometrists OR MH Pharmacists OR MH Physical Therapists OR MH Podiatry OR MH Psychologists OR MH Radiologic Technologists OR (TI (Nurs* OR optom* OR dent* OR midwif* OR pharm* OR medica* OR osteopath* OR chiropract* OR podiatr* OR occupational therap* OR paramedic* OR physiotherap* OR psycholo* OR radiograph* OR doctor* OR physician* OR general practitioner* OR health care profession* OR healthcare profession* OR physical therapist* OR early n2 career OR mid n2 career OR late n2 career)) OR (AB ( Nurs* OR optom* OR dent* OR midwif* OR pharm* OR medica* OR osteopath* OR chiropract* OR podiatr* OR occupational therap* OR paramedic* OR physiotherap* OR psycholo* OR radiograph* OR doctor* OR physician* OR general practitioner* OR health care profession* OR healthcare profession* OR physical therapist*)) |
| AND | MH accreditation OR MH education, competency-based OR MH education, continuing OR MH education, nursing, continuing OR MH education, medical, continuing OR MH learning OR MH lifelong learning OR MH mentorship OR MH problem-based learning OR MH peer review OR MH professional portfolios OR MH recertification OR MH self assessment OR MH self directed learning OR MH staff development OR (TI (competency n2 framework OR competency nj2 standards OR reflective nj2 practi* OR revalidation)) OR (AB ((competency n2 framework OR competency nj2 standards OR reflective nj2 practi* OR revalidation)) |
| AND | MH clinical competence OR MH competency assessment OR MH cultural competence OR MH disruptive behaviour OR MH healthcare errors OR MH inappropriate prescribing OR MH licensure OR MH malpractice OR MH mandatory reporting OR MH outcomes of education OR MH patient safety OR MH professional competence OR MH professional practice OR MH professionalism OR MH quality of health care OR MH scope of practice OR MH scope of practice, nursing OR MH registration OR (TI (advanced n2 practice OR authentic nj2 learning OR endorsement OR extended n2 practice OR fitness n3 practice OR knowledge n2 transfer)) OR (AB (advanced n2 practice OR authentic nj2 learning OR endorsement OR extended n2 practice OR fitness n3 practice OR knowledge n2 transfer)) |
| AND | Limit to yr=2015– April 2022 |
| AND | Limit to English language |
